# Supplementary material for: Bhalin, an Essential Cytoskeleton-Associated Protein of Trypanosoma brucei Linking TbBILBO1 of the Flagellar Pocket Collar with the Hook Complex
Source: Microorganisms. 2021 Nov 11;9(11):2334. doi: 10.3390/microorganisms9112334 (PMC8623173; doi:10.3390/microorganisms9112334)
Supplement: Supplementary file 1 [file microorganisms-09-02334-s001.zip › Broster Reix et al Supplementary figures and legends.pdf]

Figure S1

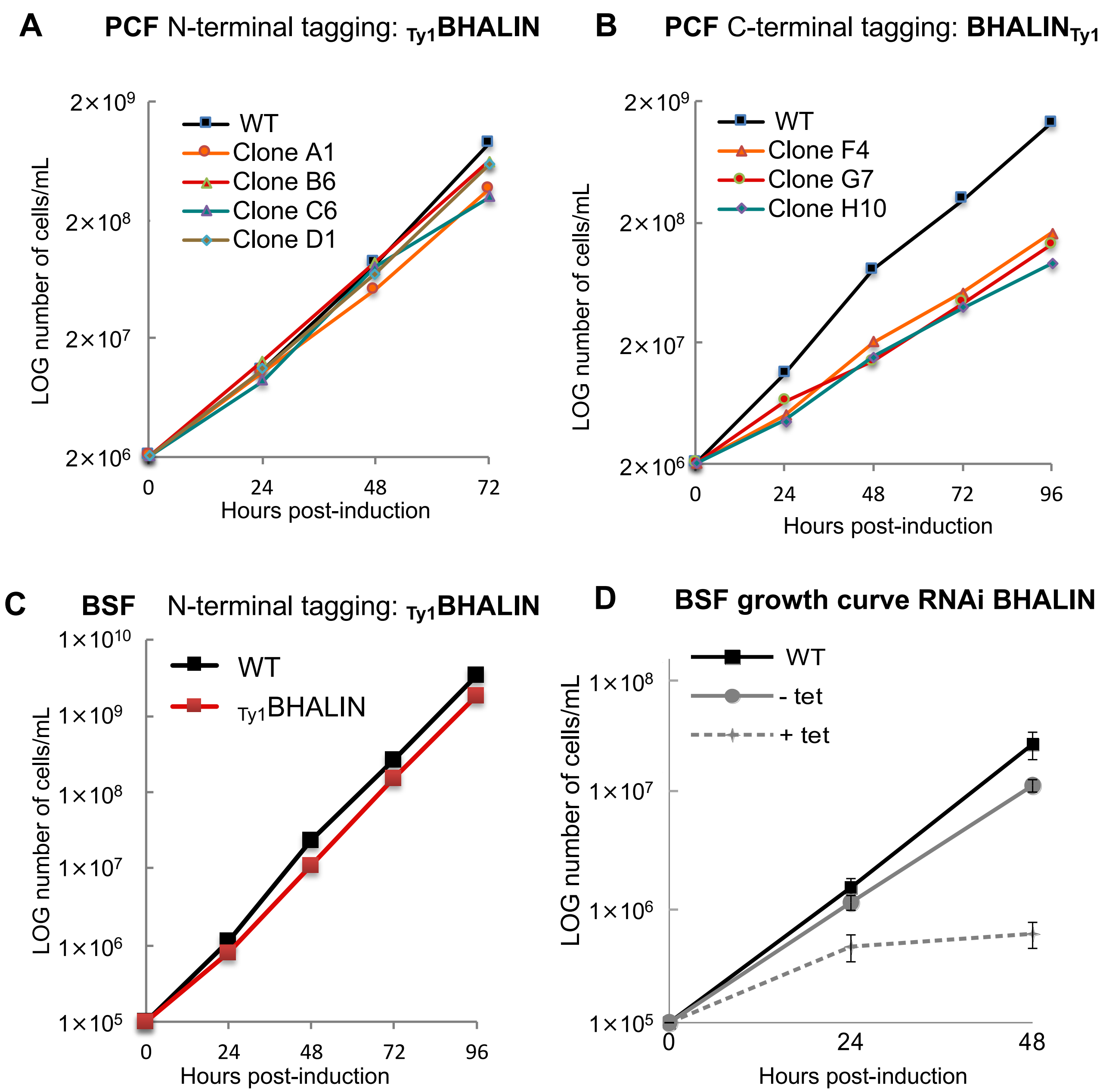
A
PCF N-terminal tagging: Ty1BHALIN
2×10^9
2×10^8
2×10^7
2×10^6
LOG number of cells/mL
WT
Clone A1
Clone B6
Clone C6
Clone D1
0
24
48
72
Hours post-induction
B
PCF C-terminal tagging: BHALINTy1
WT
Clone F4
Clone G7
Clone H10
96
C
BSF N-terminal tagging: Ty1BHALIN
1×10^10
1×10^9
1×10^8
1×10^7
1×10^6
1×10^5
Ty1BHALIN
D
BSF growth curve RNAi BHALIN
- tet
+ tet

Figure S2

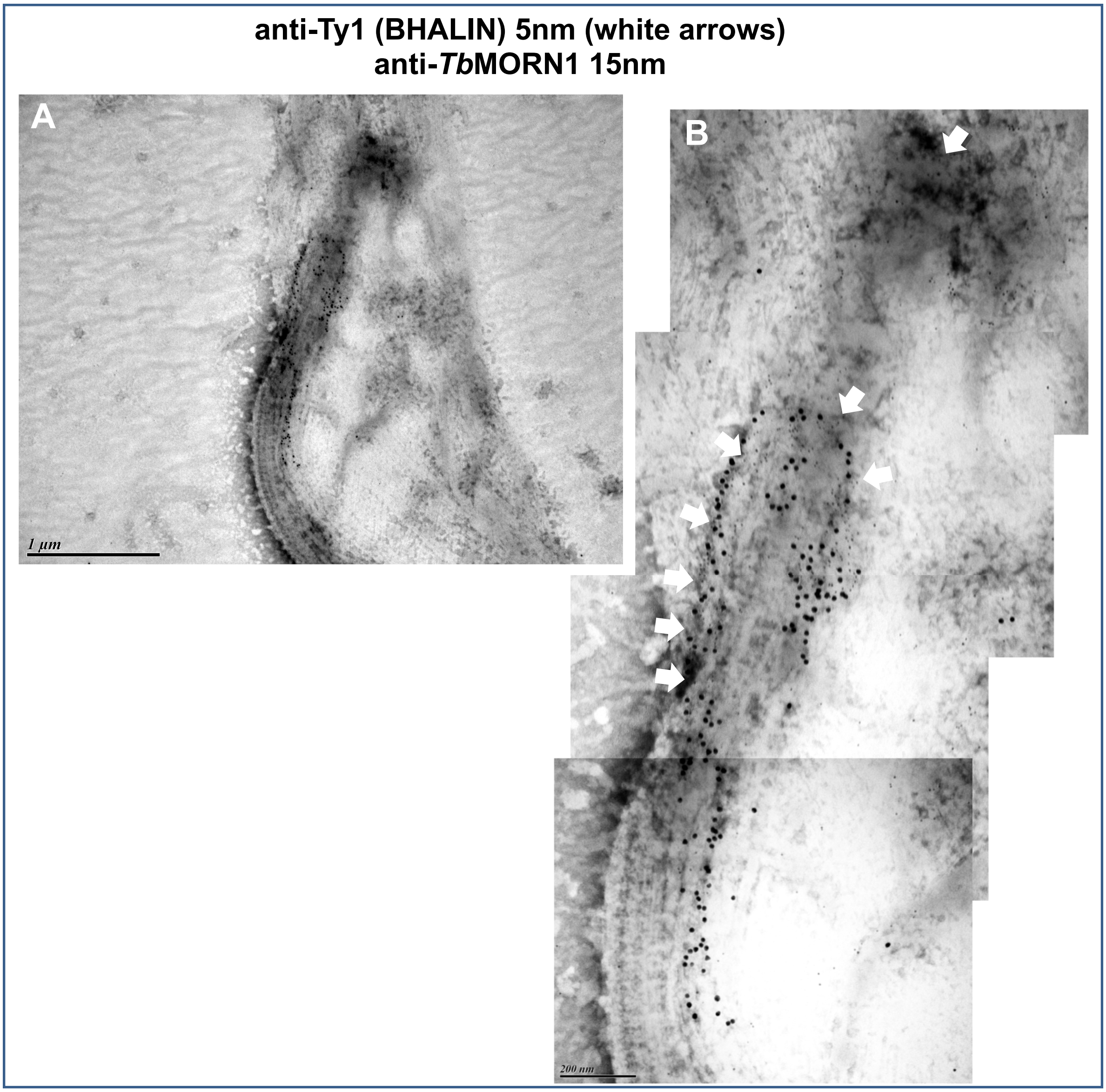
anti-Ty1 (BHALIN) 5nm (white arrows)
anti-*Tb*MORN1 15nm
A
1 µm
B
200 nm

Figure S3

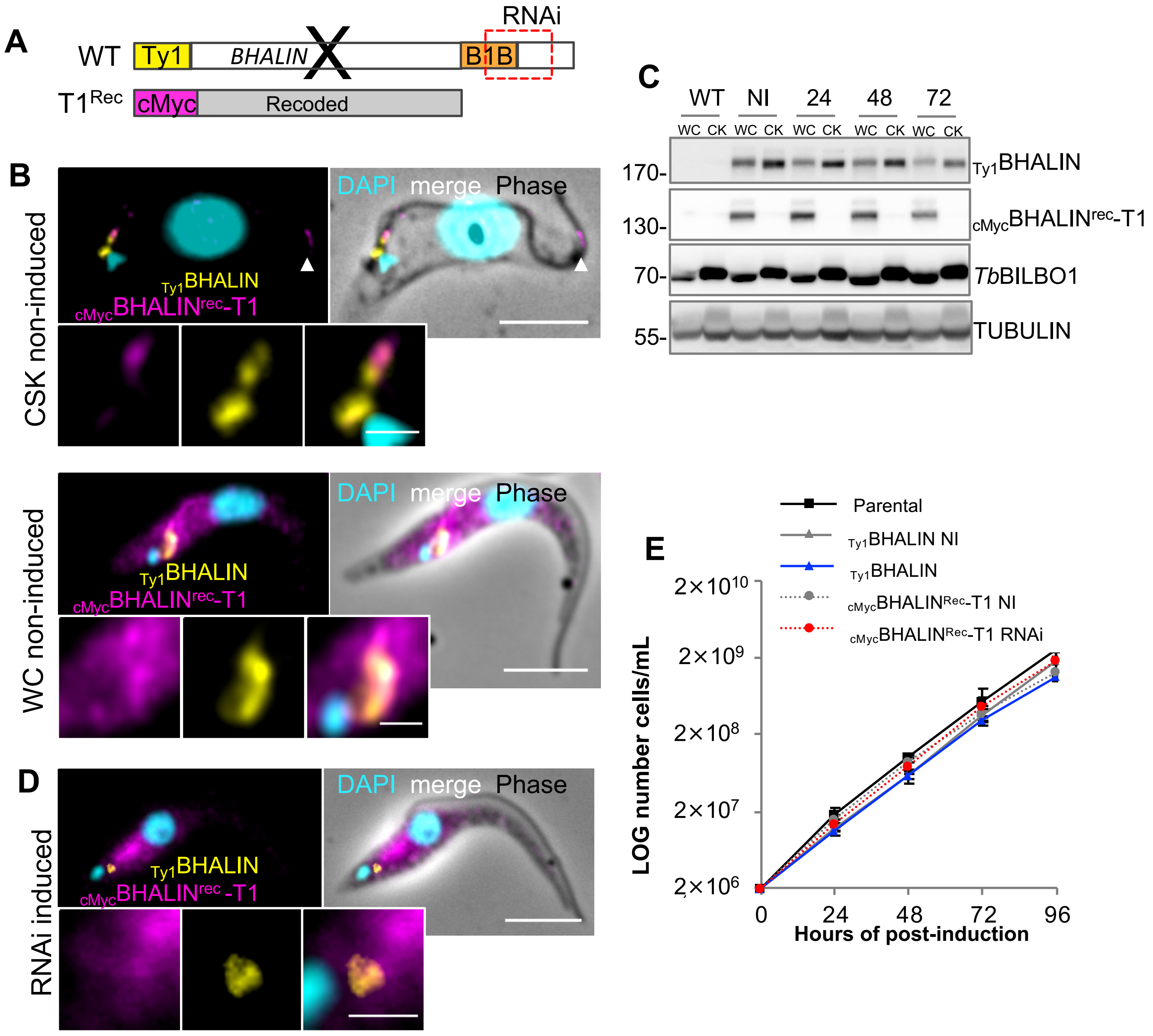

A
WT
Ty1
BHALIN
B1B
RNAi
T1Rec
cMyc
Recoded
B
CSK non-induced
DAPI merge Phase
Ty1BHALIN
cMycBHALINrec-T1
WC non-induced
DAPI merge Phase
Ty1BHALIN
cMycBHALINrec-T1
D
RNAi induced
DAPI merge Phase
Ty1BHALIN
cMycBHALINrec -T1
C
WT NI 24 48 72
WC CK WC CK WC CK WC CK WC CK
170-
130-
70-
55-
Ty1BHALIN
cMycBHALINrec-T1
TbBILBO1
TUBULIN
E
Parental
Ty1BHALIN NI
Ty1BHALIN
cMycBHALINRec-T1 NI
cMycBHALINRec-T1 RNAi
LOG number cells/mL
2×10^10
2×10^9
2×10^8
2×10^7
2×10^6
0
24
48
72
96
Hours of post-induction

Figure S4

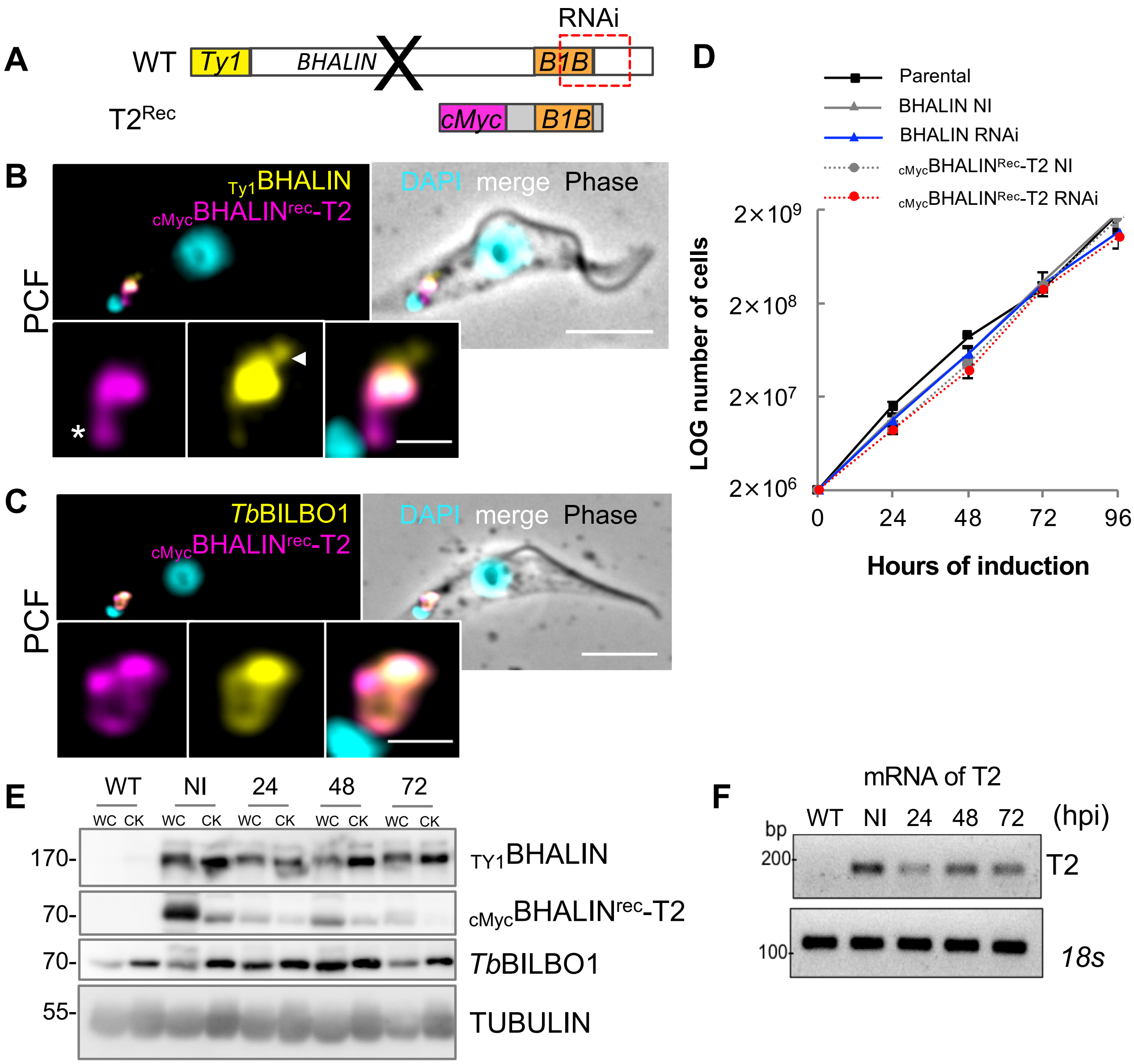
A
WT
Ty1
BHALIN
B1B
RNAi
T2Rec
cMyc
B1B
B
PCF
Ty1BHALIN
cMycBHALINrec-T2
DAPI merge Phase
C
PCF
TbBILBO1
cMycBHALINrec-T2
DAPI merge Phase
D
Parental
BHALIN NI
BHALIN RNAi
cMycBHALINRec-T2 NI
cMycBHALINRec-T2 RNAi
LOG number of cells
2×10^9
2×10^8
2×10^7
2×10^6
0
24
48
72
96
Hours of induction
E
WT NI 24 48 72
WC CK WC CK WC CK WC CK WC CK
170-
70-
70-
55-
TY1BHALIN
cMycBHALINrec-T2
TbBILBO1
TUBULIN
F
mRNA of T2
WT NI 24 48 72 (hpi)
bp
200
100
T2
18s

Figure S5

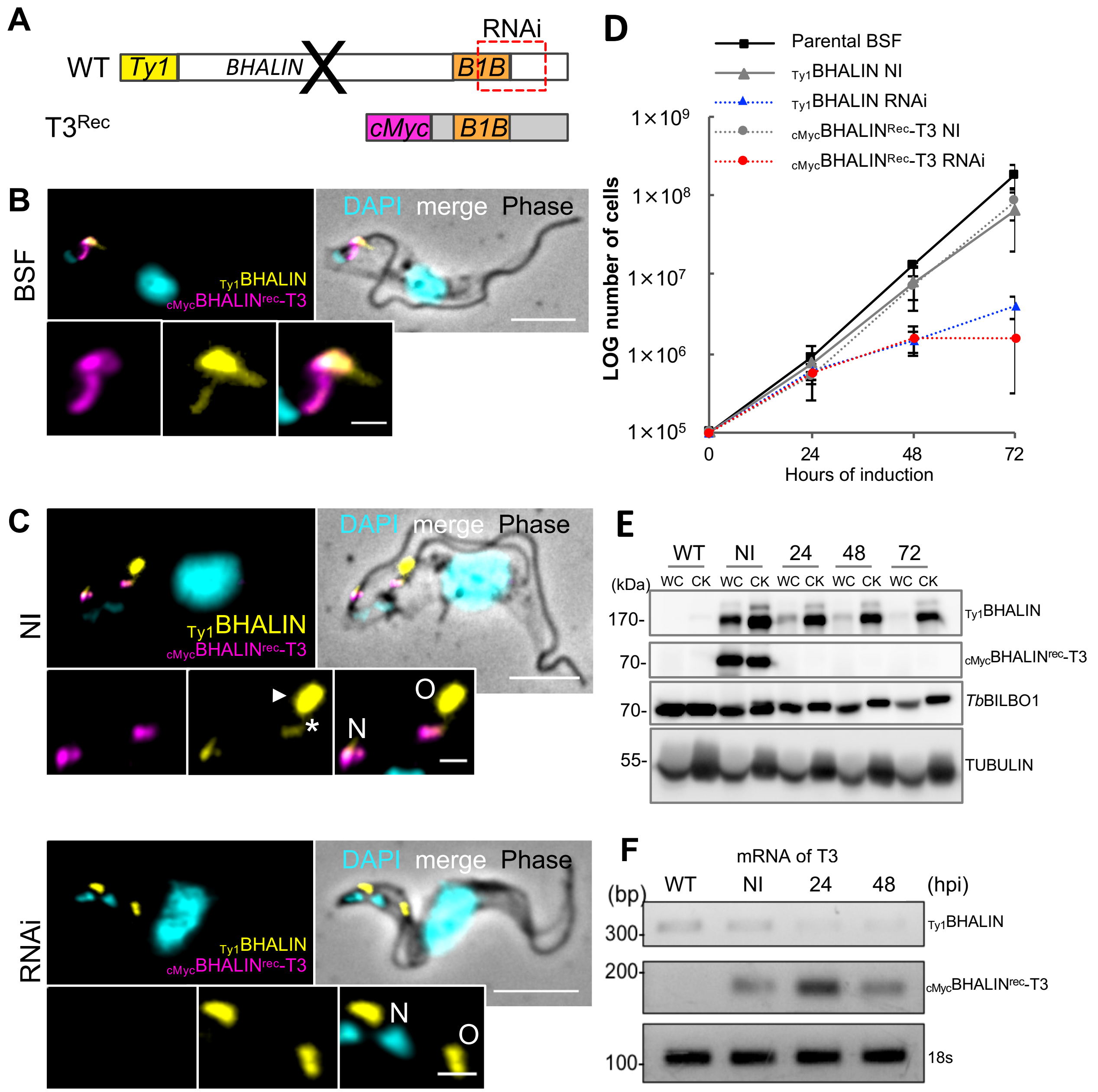

A
RNAi
WT
Ty1
BHALIN
B1B
T3Rec
cMyc
B1B
B
BSF
DAPI
merge
Phase
Ty1BHALIN
cMycBHALINrec-T3
C
NI
DAPI
merge
Phase
Ty1BHALIN
cMycBHALINrec-T3
*
N
O
RNAi
DAPI
merge
Phase
Ty1BHALIN
cMycBHALINrec-T3
N
O
D
Parental BSF
Ty1BHALIN NI
Ty1BHALIN RNAi
cMycBHALINRec-T3 NI
cMycBHALINRec-T3 RNAi
LOG number of cells
1×10^9
1×10^8
1×10^7
1×10^6
1×10^5
0
24
48
72
Hours of induction
E
WT
NI
24
48
72
(kDa)
WC CK WC CK WC CK WC CK WC CK
170-
Ty1BHALIN
70-
cMycBHALINrec-T3
70-
TbBILBO1
55-
TUBULIN
F
mRNA of T3
WT
NI
24
48
(hpi)
(bp)
300-
Ty1BHALIN
200-
cMycBHALINrec-T3
100-
18s

Figure S6

```
BHALIN_WT         AGCAAAGAGAGGTCCCTTTGTGTGGTGCAACCATGTGGGGGAAAGGTAAGTGCAAGTGAA    2100
BHALIN_recoded    AGTAAGGAACGCAGCTTGTGCGTAGTGCAACCCTGTGGAGGCAAAGTGTCGGCAAGTGAA    2100
                  ** ** **  *   * * ** ** ******** ***** ** ** **    *********

BHALIN_WT         CTAAGTGCACTGGCTTCCACGCAGCTGATGTCCAAGGACACCTCGTTGAGCGAAGACTGC    2160
BHALIN_recoded    CTATCGGCCTTGGCTTCCACTCAGTTAATGAGCAAGGATACATCGCTTTCGGAGGATTGC    2160
                  ***   **  ********** *** * ***  ****** ** *** *    ** ** ***

BHALIN_WT         GCGAGCAGGTTGCGGAAGAAGTGGGGAGTAACGCCGAAGAAGGCAGAAAAAGAGGATGAT    2220
BHALIN_recoded    GCCAGTCGACTTCGAAAGAAATGGGGTGTCACACCCAAGAAGGCCGAGAAAGAGGATGAT    2220
                  ** **  *  * ** ***** ***** ** ** ** ******** ** ************

BHALIN_WT         GAAGAAGAGAGGATACGTCGGGCAGAACGTTTTGAGAGGATCAACCTCGCGTTGCTAAAG    2280
BHALIN_recoded    GAGGAGGAACGCATTAGGCGTGCAGAAAGATTTGAGCGCATTAATCTGGCTTTGCTTAAA    2280
                  ** ** **  * **  * ** ****** * ****** * ** ** ** ** ***** **

BHALIN_WT         CTAGAAGTCTGCAGAAAGAACAATCATTCATCTCATGACACAAGGGGGTGGGCGTCACCA    2340
BHALIN_recoded    TTGGAAGTCTGTCGGAAGAATAACCACAGTTCGCACGATACGAGAGGCTGGGCATCACCC    2340
                   * ********  * ***** ** **    ** ** ** ** ** ** ***** *****

BHALIN_WT         ACAAGCGACTTGGACTCACGGTACGAAACCAAACCCTGGACTCAGGCATATTCCCCCTCT    2400
BHALIN_recoded    ACATCCGACTTAGACAGTCGTTATGAGACAAAACCGTGGACACAGGCTTACTCACCAAGT    2400
                  ***  ****** ***   ** ** ** ** ***** ***** ***** ** ** **   *

BHALIN_WT         GGTGCCACCACCGACACCCTTGACAACGGTTACACTTCATCGATTGATCCCCCACGCTCA    2460
BHALIN_recoded    GGTGCGACAACAGACACCTTGGATAATGGGTATACCTCCTCAATTGACCCGCCACGCTCT    2460
                  ***** ** ** ****** * ** ** ** ** ** ** ** ***** ** ********

BHALIN_WT         AAGTATTGGGACAGGGAAATGGCCGGAGGCACTTCTAAGAGGCATAACTCAAGACCGAGA    2520
BHALIN_recoded    AAGTACTGGGACAGAGAAATGGCGGGTGGGACGTCGAAGCGGCATAATTCACGTCCACGT    2520
                  ***** ******** ******** ** ** ** ** *** ******* *** * **  *

BHALIN_WT         TCACTGTTTCCGCCGCCGCACTCACCCGTCAAAACCTCAGCTGATCCACCGGTGGACGGC    2580
BHALIN_recoded    TCCTTGTTTCCGCCACCCCATTCTCCCGTCAAAACAAGCGCGGATCCTCCGGTCGATGGT    2580
                  **  ********** ** ** ** ***********    ** ***** ***** ** **

BHALIN_WT         CTCATTTACACACCAGCGAAAACTGCATCGGCACGCAGCGCTTCCTGGATGTTTGAAAGG    2640
BHALIN_recoded    CTTATCTACACGCCAGCTAAGACAGCCTCAGCCAGAAGTGCCTCGTGGATGTTTGAACGC    2640
                  ** ** ***** ***** ** ** ** ** **  * ** ** ** ************ *

BHALIN_WT         AGTCCTGATGAGGATAGGTACAACTCGTACTTCATGGACGCAAACTCACGGCTGCAGGAA    2700
BHALIN_recoded    AGTCCAGATGAGGATCGCTATAACAGTTACTTCATGGATGCTAATAGTCGACTGCAAGAA    2700
                  ***** ********* * ** ***   *********** ** **    ** ***** ***

BHALIN_WT         CTCCGTGTATGTGATTCAATGGCGGCGAAAAACTTTGCACGTGATCTCTCGTCGCTAGCA    2760
BHALIN_recoded    CTCCGGGTGTGTGATAGCATGGCAGCCAAAAATTTCGCGAGGGACCTTAGTAGTCTGGCG    2760
                  ***** ** ******   ***** ** ***** ** **  * ** **       ** **

BHALIN_WT         TCCGCTGTACGGGGCAGTGAACTATTTAGCATGATATCGTGA    2802
BHALIN_recoded    TCCGCCGTTCGAGGCAGTGAGCTCTTCAGTATGATTTCATAA    2802
                  ***** ** ** ******** ** ** ** ***** ** * *
```

Broster Reix *et. al*, supplementary data; figure legends

Figure S1. Growth curves of N- and C-terminal tagged BHALIN with 10xTy1 in PCF and BSF. (**A**) Growth curve of 4 clones of PCF with BHALIN tagged 10xTy1 on the N-terminus. (**B**) Growth curves of 3 clones of PCF with BHALIN tagged 10xTy1 on the C-terminus. (**C**) Growth curves of WT and of BSF tagged 10xTy1 on the N-terminus. (**D**) Growth curve for the WT, non-induced (-tet) and BHALIN RNAi-induced (+tet) cells.

Figure S2. Immuno-electron micrographs of PCF detergent-extracted cells showing co-localisation of BHALIN and *Tb*MORN1. (**A**) Posterior end of a PCF trypanosome showing the microtubule corset outlining the cell body and the darker impression of the flagellum. (**B**) Magnification of (**A**), where images have been overlaid. White arrows indicating the co-localisation of labelling of 15 nm gold beads against *Tb*MORN1 and 5 nm gold beads against Ty1-tagged BHALIN.

Figure S3. The N-terminus of BHALIN (T1) is cytoplasmic in trypanosomes. (**A**) Schematic drawing to illustrate WT BHALIN and recoded $_{cMyc}$BHALIN^{rec}-T1. (**B**) IFA of PCF detergent-extracted (CSK) or whole cell (WC) trypanosomes expressing $_{Ty1}$BHALIN (yellow) and $_{cMyc}$BHALIN^{rec}-T1 (magenta). (**C**) Western blot of whole cell (WC) and detergent-extracted cytoskeletons (CK) from WT cell line, NI and at 24 h intervals of post-induction. $_{Ty1}$BHALIN probed with anti-Ty1 in the first row and, $_{cMyc}$BHALIN^{rec}-T1 probed with anti-cMyc in the second row. *Tb*BILBO1 probed with anti-BILBO1 (third row). Tubulin was used as a loading control. (**D**) Whole cell IFA showing the cytoplasmic pool of $_{cMyc}$BHALIN^{rec}-T1 (magenta) after RNAi knockdown for 24 h. (**E**) Growth curve of Parental (the original non-modified or tagged cell line), the RNAi BHALIN cell line and the $_{Ty1}$BHALIN/ $_{cMyc}$BHALIN^{rec}-T1 expressing cell line. Scale bars in B and D represent 5 μm, insets 1 μm.

Figure S4. The *Tb*BILBO1-binding domain (B1B) of BHALIN co-localises with *Tb*BILBO1 in vivo. (**A**) Schematic drawing to illustrate WT and $_{cMyc}$BHALIN^{rec}-T2. (**B**) A detergent extracted PCF trypanosome labelled with anti-cMyc for $_{cMyc}$BHALIN^{rec}-T2 and anti-Ty1 for $_{Ty1}$BHALIN. The arrowhead indicates the shank of the hook; the asterisk indicates labelling between the FPC and the basal bodies. These signals are shown because they are often difficult to visualise. (**C**) IFA image of a PCF cytoskeleton labelled with anti-cMyc to localise $_{cMyc}$BHALIN^{rec}-T2 (magenta) and anti-*Tb*BILBO1 (yellow) on the FPC. Scale bars in B and C represent 5 μm, inset 1 μm. (**D**) Growth curve of parental, the RNAi BHALIN cell line and the cell line with one allele of *BHALIN* replaced with a truncated recoded sequence of the *Tb*BILBO1-binding domain ($_{cMyc}$BHALIN^{rec}-T2), before (NI) and after (RNAi) induction. (**E**) Western blot of whole cell (W) and detergent-extracted cytoskeleton (CK) trypanosome samples from WT, RNAi NI and RNAi induced in the $_{cMyc}$BHALIN^{rec}-T2 cell line. First row $_{Ty1}$BHALIN probed with anti-Ty1 which shows a small reduction in protein levels followed by an increase to near normal levels. Second row probed with anti-cMyc for $_{cMyc}$BHALIN^{rec}-T2. Tubulin as loading control. (**F**) Levels of mRNA of $_{cMyc}$BHALIN^{rec}-T2 after BHALIN knockdown in PCF showing that $_{cMyc}$BHALIN^{rec}-T2 is resistant to RNAi knockdown.

Figure S5. BHALIN-T3 cannot rescue the RNAi lethal phenotype in BSF. (**A**) Schematic drawing to illustrate WT and recoded T3. (**B**) IFA image of a 1K1N BSF cytoskeleton labelled anti-cMyc to detect $_{cMyc}$BHALIN^{rec}-T3, (magenta) and anti-Ty1 labelling of $_{Ty1}$BHALIN (yellow). (**C**, upper panel) A 2K1N BSF cytoskeleton, non-induced, labelled as in (**B**), O = old FPC/HC and N = new FPC/HC. (**C**, lower panel) IFA of a BSF trypanosome cytoskeleton induced after 24 h RNAi knockdown (RNAi) and labelled as above. The labelling for the $_{Ty1}$BHALIN (yellow) shows that the protein had not yet been completely eliminated, whilst there was no labelling for T3. Scale bars in B and C represent 5 μm, inset 1 μm. (**D**) Growth curve for parental, $_{Ty1}$BHALIN expressing and $_{Ty1}$BHALIN + $_{cMyc}$BHALIN^{rec}-T3 expressing cell lines inducible for BHALIN RNAi, Non-induced (NI) and RNAi induced. (**E**) Western blot of whole cell (WC) and cytoskeletons (CK) samples from WT cells and $_{Ty1}$BHALIN + $_{cMyc}$BHALIN^{rec}-T3 expressing cell lines inducible for BHALIN RNAi. First row probed with anti-Ty1 showing the $_{Ty1}$BHALIN protein (WT allele). The second row probed with anti-cMyc showing the T3 protein. Tubulin is loading control. (**F**) Levels of *BHALIN* and *$_{cMyc}$BHALIN^{rec}-T3* mRNA after knockdown in BSF showing reduced levels of mRNA of $_{Ty1}$BHALIN but not *BHALIN^{rec}-T3*.

Figure S6. CLUSTAL Omega nucleotide sequence alignment of BHALIN WT gene vs. recoded BHALIN of the RNAi target. The BHALIN wild-type gene is displayed in the upper lanes and recoded gene in the lower lane. The area highlighted in yellow (nucleotides 2048-2753) indicates the region targeted for RNAi knockdown.
